# Effects of surface sterilization of fertile eggs on the yolk microbiota during the chicken embryo development

**DOI:** 10.3389/fvets.2024.1493415

**Published:** 2024-11-27

**Authors:** Peng Ding, Xi He, Minxi Li, Sai Jiang, Yanmei Peng

**Affiliations:** ^1^Institute of Innovative Traditional Chinese Medications, Hunan Academy of Chinese Medicine, Hunan, China; ^2^Furong Laboratory, Changsha, Hunan, China; ^3^College of Animal Science and Technology, Hunan Agricultural University, Changsha, Hunan, China

**Keywords:** eggshell sterilization, embryogenesis, *Staphylococcus saprophyticus*, microbial colonization, immunity

## Abstract

Surface sterilization of the fertile eggs is a common process for commercial broiler breeding to avoid pathogenic bacterial infections before incubation. However, it is also possible to remove the beneficial microbes that might contribute to the development of chicken embryos. Thus, we established a model to mimic surface sterilization in the laboratory by rubbing fertile eggs with 70% ethanol and investigated the effect of eggshell surface sterilization on the yolk microbiota and its potential role in chicken (*Gallus gallus*) embryo development. In total, 460 Ross 308 fertile eggs were randomly divided equally into the eggshell surface sterilized group (CS, commercial egg sterilization group) and the eggshell surface unsterilized group (CC, commercial egg control group). The shell surface of group CS was sterilized with 70% alcohol before incubation (E0, embryonic stage), while that of group CC was not sterilized before incubation. At each sampling day (E0, E07, E15, and E21), 24 fertile eggs from each of the two groups were randomly selected to collect the yolk samples and weigh the embryos. The results showed that the surface sterilization of eggshells before incubation improved the development of chicken embryos from E15 to E21 but reduced the diversity of the yolk microbiota. In the whole process of embryogenesis, the relative abundance of Firmicutes, Bacteroidetes, and Actinobacteria in the egg yolk of group CS was lower than that of group CC before incubation. Indeed, the surface sterilization of fertile eggs significantly reduced the relative abundance of *Staphylococcus saprophyticus* and other pathogenic bacteria in the yolk, which may result in the better development of chicken embryos.

## Introduction

Fertile eggs of commercial broilers were commonly sterilized before incubation by fumigation to prevent infection from pathogenic bacteria that come from the microbes in the maternal oviduct or hindgut (1, 2). However, the bacteria that reside on the shell surface of fertile eggs are a large community of microbial populations that play a role in shaping the microbiota of the embryo and even the intestine of newly hatched birds (3). Early studies showed that surface sterilization did not affect the hatchability of fertilized eggs but affected the microbial composition of the fertilized eggs (4, 5). In addition, a study compared different fumigation or spraying methods for fertile egg sanitization, but the results showed that the disinfected group did not influence the microbiota composition of the yolk sac compared to the non-disinfected group (6). Thus, it is still controversial about whether the proper cleaning procedure of the fertile eggs would be beneficial for the colonization of microbes in chicken embryos.

Microorganisms present during the embryonic development of animals have been proven to serve a great function in interacting with embryonic growth by modulating immune responses and nutrient exchanges (7, 8). Embryogenesis in avian species is largely determined by the nutrients and microbiota deposited in the eggs, as they develop separately from the maternal surrogate. Previous studies have shown that the administration of antibiotics in the diet of breeder hens would result in a decreased growth-promoting effect of the antibiotics in post-hatch birds (9, 10). In addition, *in ovo* delivery of prebiotics or synbiotics to developing chicken embryos was able to boost gut health and body immunity in post-hatch chickens (11, 12). Our published data identified several microbial metabolites in egg yolk during embryonic development, which might indicate that microorganisms in fertile eggs are probably involved in nutrient metabolism for embryonic growth (13). Therefore, it is worthwhile to reconsider the effects of fertile egg sanitization not only on the elimination of anaerobic pathogenic bacteria but also on the unexpected loss of the bacteria, which might be beneficial to chicken embryo development.

In this study, we simply stimulated the surface sterilization procedure by using 70% alcohol to clean the fertile eggshell before incubation and explored the effect of eggshell surface sterilization on the microbiota within the egg yolk and its potential role in the development of the chicken embryo.

## Materials and methods

### Animals and sample preparation

A total of 460 undamaged and uniformly sized fertile eggs of Ross 308 commercial broilers were purchased from the Shuncheng Broiler Breeder Farm (Ningxiang, Hunan). All eggs were collected from 35-week-old flocks. The animal procedures in this study were approved by the Institutional Animal Care and Use Committee of Hunan Agricultural University (GBT2018). The fertile eggs were randomly divided equally into group CS and group CC and the initial weight was recorded. There was no significant difference in the initial egg weight between the two groups of fertile eggs (CS: 68.37 ± 3.75 g, CC: 67.81 ± 4.17 g, *p* = 0.135, *n* = 460). Before incubation at 37 ± 0.5°C with 60 ± 5% relative humidity, the shell surface of group CS was sterilized with 70% alcohol, while that of group CC was not sterilized. The alcohol was sprayed evenly over the entire surface of the egg and then wiped clean with sterile cotton. After sterilization of each egg, the gloves were sterilized with alcohol, followed by the surface sterilization of the eggshell of the next egg. A sterile marker (DOTCH Puru Marker) was used to number all eggs. The incubators were sterilized by ultraviolet light sanitization before use, and the two groups were separated. Eggs were transferred to pedigree hatch baskets at E18. Each egg was isolated from the others in individual cells in the hatcher tray, thus retaining the treatment information of each chick upon fertilization. All procedures were performed in a sterile room with all the manipulations in a sterile hood. The ventilation system was connected to the common environmental air supply without any sterilizing treatments. All the eggs were candled at E05 to check the fertility and the unfertilized eggs were eliminated. In total, 24 fertile eggs from each of the two groups were randomly selected to collect samples (E0, E07, E15, and E21) and to weigh the embryos at the indicated time points (E07, E15, and E21) (3), respectively. Chicken embryo growth performance was measured by calculating the ratio of chicken embryo weight to initial egg weight. In total, 12 of these 24 eggs were selected to investigate the microbiota in the egg yolk. The eggshell was gently peeled off using a sterile tweezer to expose the embryo, and then, the yolk samples (5 mL) were collected by puncturing the yolk sac membrane with a syringe and homogenized. Subsequently, the abdomens of chicken embryos were dissected with a scalpel and then the whole gut samples were carefully collected on a 4°C sanitized working bench. The yolk samples and gut samples were stored at –80°C after snap freezing in the liquid nitrogen. After hatching, the number of hatched chickens was recorded to determine the hatchability with the following formula: Hatchability = (No. of hatched eggs/No. of fertile eggs after sampling) × 100.

### DNA extraction and 16 s rDNA sequencing

Approximately 5 mL of egg yolk samples were thawed on ice, then put in 10 mL of sterile 1× PBS buffer to remove the excessive yolk fat, and centrifuged at 16000 rpm for 5 min, and DNA was extracted from yolk samples using the TIANamp Micro DNA Kit (TIANGEN, cat#DP316). Total DNA was extracted from homogenized embryonic intestinal samples using the TIANamp Stool DNA Kit (TIANGEN, cat#DP328), according to the manufacturer’s instructions. The concentration and quality of the isolated DNA were assessed by using a NanoDrop spectrophotometer (Thermo Scientific, United States). Amplicons of the V4 and V5 hypervariable regions of 16S rRNA were amplified by using the sample-specific sequence barcoded fusion primers (forward 5’-GTGCCAGCMGCCGCGGTAA-3’ and reverse 5’-CCGTCAATTCMTTTRAGTTT-3’). Sample-specific 7-bp barcodes were incorporated into the primers for multiplex sequencing. The PCR components contained 5 μL of Q5 reaction buffer (5×), 5 μL of Q5 High-Fidelity GC buffer (5×), 0.25 μL of Q5 High-Fidelity DNA Polymerase (5 U/μL), 2 μL (2.5 mM) of dNTPs, 1 μL (10 uM) of each forward and reverse primer, 2 μL of DNA template, and 8.75 μL of ddH_2_O. Thermal cycling consisted of an initial denaturation at 98°C for 2 min, followed by 35 cycles consisting of denaturation at 98°C for 15 s, annealing at 55°C for 30 s, and extension at 72°C for 30 s, with a final extension of 5 min at 72°C. PCR amplicons were purified with Agencourt AMPure Beads (Beckman Coulter, Indianapolis, IN) and quantified using the PicoGreen dsDNA Assay Kit (Invitrogen, Carlsbad, CA, USA). 16S rRNA sequencing was performed using the Illumina Novaseq_PE250 (Illumina) sequencing platform. Sequencing services were provided by Personal Biotechnology Co., Ltd., Shanghai, China. The data were analyzed by using the free online platform Personalbio GenesCloud^1^ and a software package for analyzing taxonomic or metabolic profiles (STAMP v2.1.3).

### Sequence analysis

In total, 13,356,685 high-quality reads were generated for further analysis. The Quantitative Insights Into Microbial Ecology (QIIME2, 2019.4) pipeline was used to process the sequencing data, as previously described (14). Briefly, raw sequencing reads with exact matches to the barcodes were assigned to the respective samples and identified as valid sequences. The low-quality sequences were filtered through the following criteria (15, 16): sequences that had a length of <150 bp, sequences that had average Phred scores of <20, sequences that contained ambiguous bases, and sequences that contained mononucleotide repeats of >8 bp. Paired-end reads were assembled using FLASH (17). After chimera detection, the remaining high-quality sequences were clustered into amplicon sequence variants (ASVs) with 97% sequence identity using UCLUST. A representative sequence was selected from each ASV using default parameters. ASV taxonomic classification was conducted by BLAST searching the representative sequences set against the Greengenes database (18, 60) using the best hit (19). An ASV table was further generated to record the abundance of each ASV in each sample and the taxonomy of these ASVs. ASVs containing less than 0.001% of the total sequences across all samples were discarded. To minimize the difference in sequencing depth across samples, an averaged, rounded, rarefied ASV table was generated by averaging 100 evenly resampled ASV subsets below 90% of the minimum sequencing depth for further analysis.

### Bioinformatics and statistical analysis

Sequence data analyses were mainly performed using QIIME and R (v3.2.0) packages. ASV-level alpha diversity indices, such as the Chao1 richness estimator, abundance-based coverage estimator (ACE) metric, Shannon diversity index, and Simpson index, were calculated using the ASV table in QIIME (20, 61). ASV-level ranked abundance curves were generated to compare the richness and evenness of ASVs among samples. Beta diversity analysis was performed to investigate the structural variation of microbial communities across samples using non-metric multidimensional scaling (NMDS) and the unweighted pair-group method with arithmetic means (UPGMA) hierarchical clustering (21). Differences in the UniFrac distances for pairwise comparisons between groups were determined using Student’s *t*-test and the Monte Carlo permutation test with 1,000 permutations and visualized through box-and-whiskers plots. Principal coordinate analysis (PCoA) was also conducted based on the genus-level compositional profiles (21). The significance of microbiota structure differentiation between groups was assessed by permutational multivariate analysis of variance (PERMANOVA) (22) an analysis of similarities (ANOSIM) (23, 24) using the R package “vegan.” The taxonomy composition and abundance were visualized using MEGAN (62) and GraPhlAn (25). The R package “VennDiagram” was used to generate Venn diagrams to visualize shared and unique ASVs between samples based on the occurrence of ASVs between samples, regardless of their relative abundance (26). Taxa abundances at the phylum, class, order, family, and genus levels were statistically compared between samples or groups by Metastats (27) and visualized as violin plots. Linear discriminant analysis effect size (LEfSe) was performed to detect differentially abundant taxa between groups using the default parameters (28). Partial least squares discriminant analysis (PLS-DA) was also introduced as a supervised model to reveal the microbiota variation between groups, using the “plsda” function in the R package “mixOmics” (29). Microbial functions were predicted by phylogenetic investigation of communities by reconstruction of unobserved states (PICRUSt) based on high-quality sequences (30). *T*-test after inverse sine transformation of ratio data, with a *p*-value of <0.05 as the criterion for significant difference.

### Culture and identification of *Staphylococcus*

A selective medium (Baird-Parker agar base, Hope Bio-Technology (Qingdao) Co., Ltd.) was used to isolate and culture *Staphylococcus* from the yolk. The yolks from the two groups were directly coated on the plate medium, placed in a biochemical incubator, and incubated for 48 h at 37°C, respectively. DNA was extracted from the colonies in the culture medium using the Ezup Column Bacteria Genomic DNA Purification Kit (Sangon Biotech Co., Ltd., Shanghai). Sequencing was performed after amplification by PCR, followed by comparative analysis on the NCBI ribosome database (Table 1).

**Table 1:** The identification of *Staphylococcus* strain.

Description	Max score	Total score	Query cover	Per. Ident	Acc. Len	Accession
*Staphylococcus saprophyticus* strain FDAARGOS_336 chromosome, complete genome	2,684	16,028	100%	99.93%	2,578,483	CP022056.2
*Staphylococcus saprophyticus* strain IARI-BGL 14 16S ribosomal RNA gene, partial sequence	2,684	2,684	100%	99.93%	1,514	KT441038.1
*Staphylococcus pseudoxylosus* strain Colony292 chromosome	2,684	18,740	100%	99.93%	2,910,105	CP075501.1
*Staphylococcus saprophyticus* strain IARI-ABR-2 16S ribosomal RNA gene, partial sequence	2,684	2,684	100%	99.93%	1,470	JX428964.1
*Staphylococcus saprophyticus* strain AT7 16S ribosomal RNA gene, partial sequence	2,684	2,684	100%	99.93%	1,519	GU097199.1
*Staphylococcus saprophyticus* strain UTI-045 chromosome, complete genome	2,678	15,998	100%	99.86%	2,639,876	CP054831.1
*Staphylococcus saprophyticus* strain UTI-058y chromosome, complete genome	2,678	16,013	100%	99.86%	2,550,339	CP054440.1
*Staphylococcus saprophyticus* strain UTI-056 chromosome, complete genome	2,678	16,007	100%	99.86%	2,553,871	CP054444.1
*Staphylococcus saprophyticus* strain UTI-035 chromosome, complete genome	2,678	16,041	100%	99.86%	2,569,353	CP054434.1

## Results

### Changes in chicken embryo development and yolk microbiota due to eggshell surface sterilization

**Figure 1:**
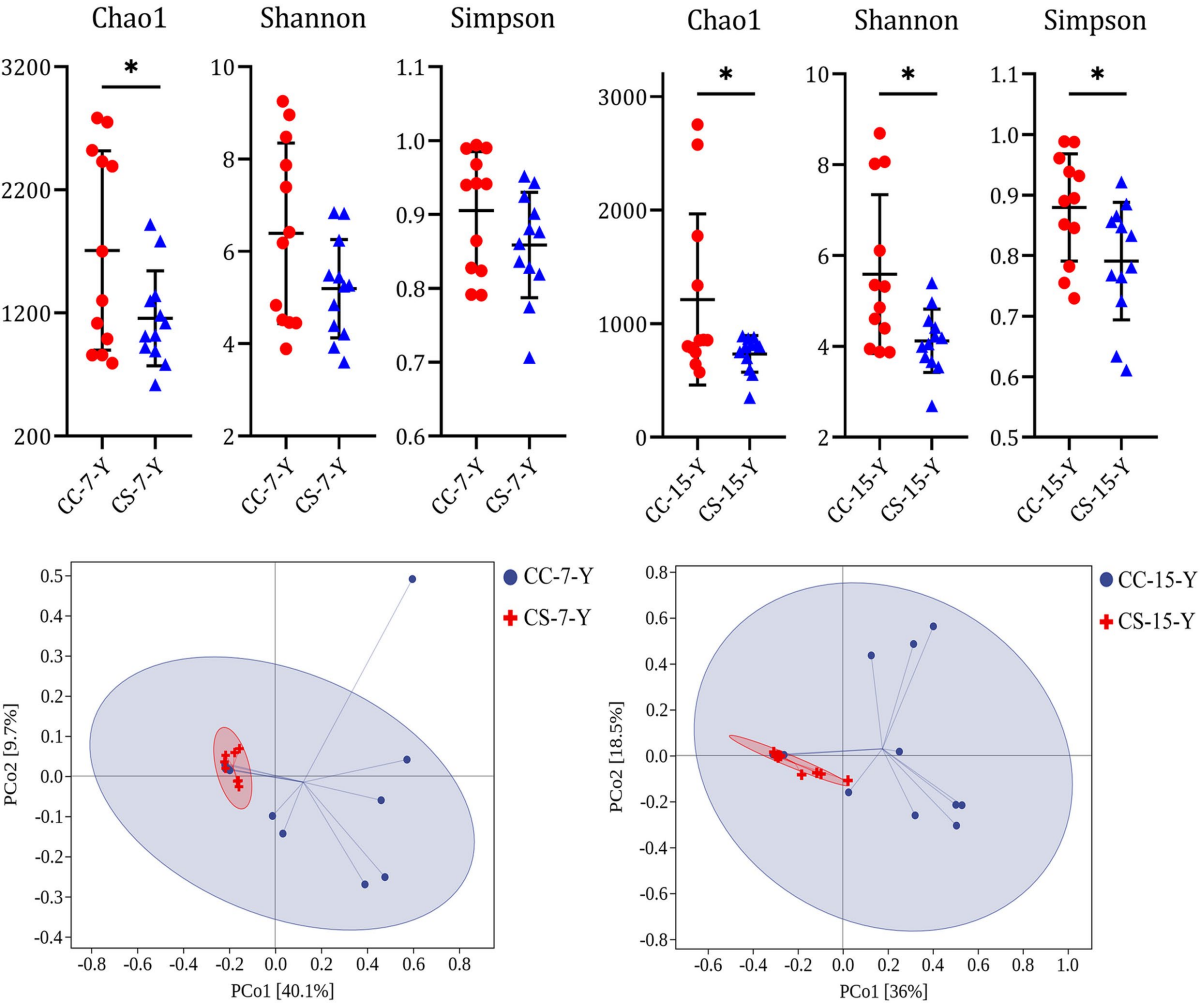
Effect of eggshell surface sterilization before incubation on the microbial diversity in the yolk of fertile eggs. CC represents commercial broiler breeder eggs without eggshell surface sterilization, CS represents commercial broiler breeder eggs with eggshell surface sterilization, Y represents yolk, and the number represents the days of incubation. Alpha diversity of yolk microbiota. Beta diversity of yolk microbiota.

The results showed that the ratio of embryo weight to initial egg weight of group CS was similar to group CC at E7 and E15, but group CS was significantly higher than group CC at E21 (Table 2, *p* = 0.002, *n* = 48), and the weight of hatched chicks in group CS was also increased compared to group CC (*p* = 0.069, *n* = 48). Fertility (*p* = 0.549, *n* = 239), hatchability (*p* = 0.230, *n* = 229), and embryonic mortality (*p* = 0.307, *n* = 229) did not differ between treatments. To examine the effect of eggshell surface sterilization on the yolk microbiota within fertile eggs, we analyzed the yolk microbial diversity of fertile eggs in the two groups at each sampling time point. The results showed that the alpha diversity of the yolk microbiota in group CS was lower than that of group CC at E07 (Figure 1A) and E15 (Figure 1B), and the difference in all indices between the two groups was significant at E15 (*p* < 0.050). The PCoA results indicated that the beta diversity of the yolk microbiota in the two groups was similar at the early stages of incubation (Supplementary Figure S1D). However, the clustering of beta diversity of the yolk microbiota was more concentrated in group CS than in group CC at E07 (Figure 1C) and E15 (Figure 1D). By the day the chicken hatched, the diversity of the microbiota in the yolk and gut tended to be the same between the two groups (Supplementary Figures S1E,F).

**Table 2:** Effect of eggshell surface sterilization on the growth of chicken embryos and hatchability.

Items		Control	Sterilization group	*P*-value^*^
E7	Egg weight (g)	68.88 ± 3.43	67.95 ± 5.84	0.636
	Embryo weight (g)	0.82 ± 0.05	0.85 ± 0.17	0.535
	Embryo weight/Egg weight (%)	1.19 ± 0.08	1.26 ± 0.26	0.397
E15	Egg weight (g)	69.06 ± 2.43	69.48 ± 3.55	0.733
	Embryo weight (g)	17.22 ± 1.83	17.69 ± 1.67	0.518
	Embryo weight/Egg weight (%)	24.92 ± 2.47	25.44 ± 1.96	0.573
E21	Egg weight (g)	66.48 ± 3.46	64.69 ± 3.00	0.189
	Chick weight (g)	46.66 ± 2.68	49.38 ± 4.15	0.069
	Chick weight/Egg weight (%)	70.28 ± 4.27^b^	76.26 ± 4.29^a^	0.002
	Fertility (%)	95.83 ± 0.43	95.80 ± 0.47	0.549
	Hatchability of set eggs (%)	87.50 ± 1.33	88.24 ± 1.24	0.230
	Hatchability of fertile eggs (%)	91.70 ± 1.20	92.10 ± 1.11	0.307

In the same row, values with no letter or the same letter superscripts means no significant difference (*p* > 0.05), while with different letter superscripts mean significant difference (*p* < 0.05).

### Changes in chicken embryo microbiota at different developmental stages

**Figure 2:**
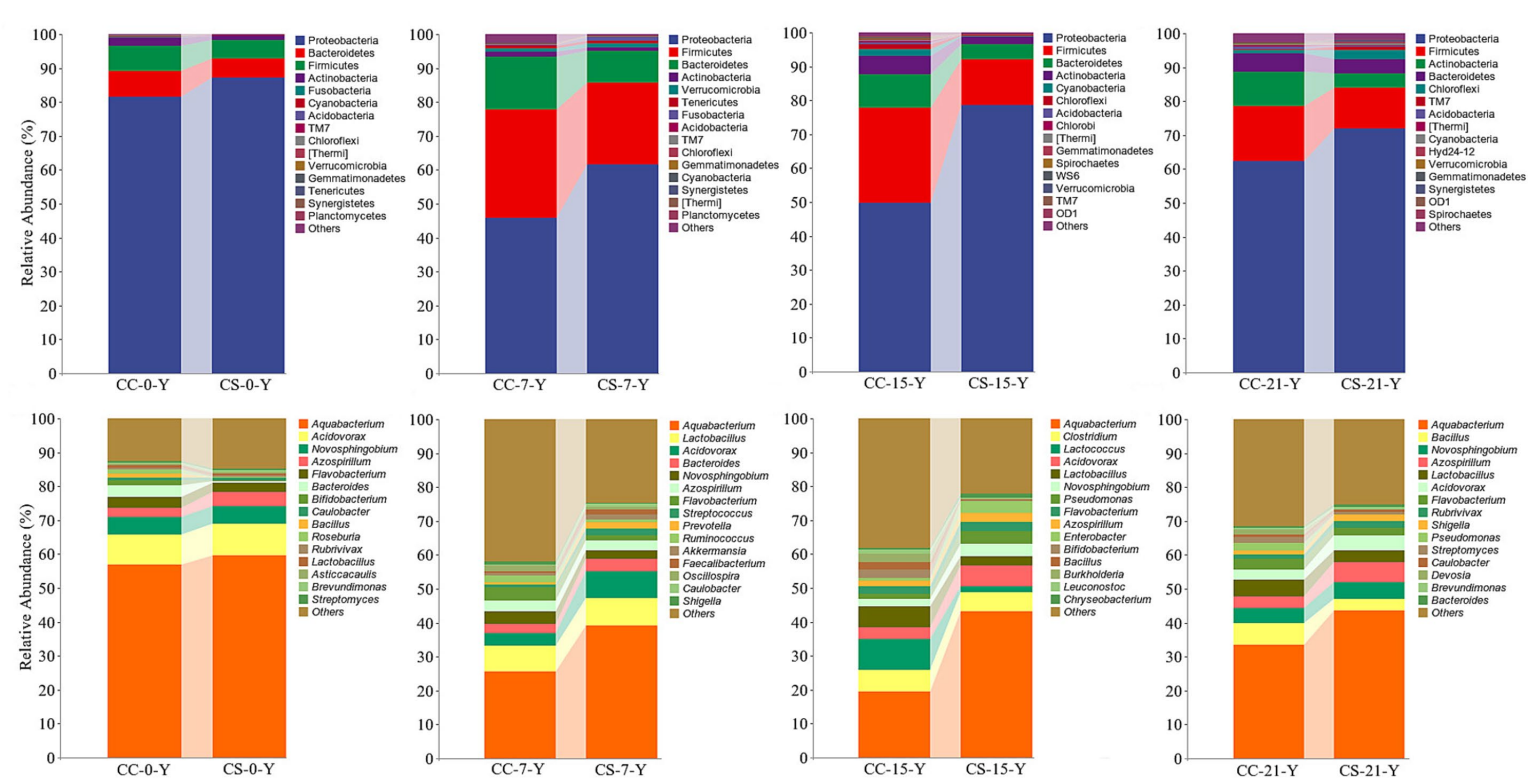
Relative abundance of yolk microbiota in eggshell surface sterilized and unsterilized fertile eggs during the embryogenesis. CC represents commercial broiler breeder eggs without eggshell surface sterilization, CS represents commercial broiler breeder eggs with eggshell surface sterilization, Y represents yolk, and the number represents incubation days. At the phylum level. At the genus level.

To analyze the changes in embryo microbiota in the two groups, we compared the relative abundance of the yolk microbiota at different time points, respectively. The results showed that Proteobacteria, Firmicutes, and Bacteroidetes were dominant at the phylum level at all different stages, followed by Actinobacteria and Chloroflexi. The yolk microbiota affected by eggshell surface sterilization is mainly dominated on Proteobacteria and Firmicutes at the phylum level (Figure 2). Notably, the abundance of Proteobacteria in the yolk of the eggshell sterilization group (49.65%) was significantly lower than that of the non-sterilization group (78.53%) at E15 (Supplementary Table S1, *p* = 0.003, *n* = 24), while becoming similar at E21 (*p* = 0.262, *n* = 24). The abundance of Bacteroidetes in the yolk of eggs with shell sterilization was lower than that of eggs without shell sterilization during embryogenesis (Figure 2), and the same trend was observed in the intestines of hatched chicks (Supplementary Figure S2). At the genus level, *Aquabacterium*, *Acidovorax*, *Lactobacillus*, *Novosphingobium*, and *Azospirillum* were the dominant bacteria in the yolk (Figure 2), while *Prevotella*, *Bacteroides*, *Faecalibacterium*, and *Clostridium* were the dominant bacteria in the intestine (Supplementary Figure S2B). The abundance of *Aquabacterium* in the yolk of fertile eggs from the surface sterilization group was higher than that of the unsterilized group during the embryogenesis, and the difference reached a significant level at E15 (Supplementary Table S2, *p* = 0.012, *n* = 24). The changes in the relative abundance of *Acidovorax* in the yolk between the two test groups at each developmental stage were similar to those of *Aquabacterium*. Meanwhile, we found that *Lactococcus* in the yolk of fertile eggs with surface sterilization was clearly lower than that of the unsterilized eggs at E15 (Supplementary Table S2, *p* = 0.082, *n* = 24) and with negligible levels at other time points.

### Filtration and analysis of the differential microbiota in the yolk

**Figure 3:**
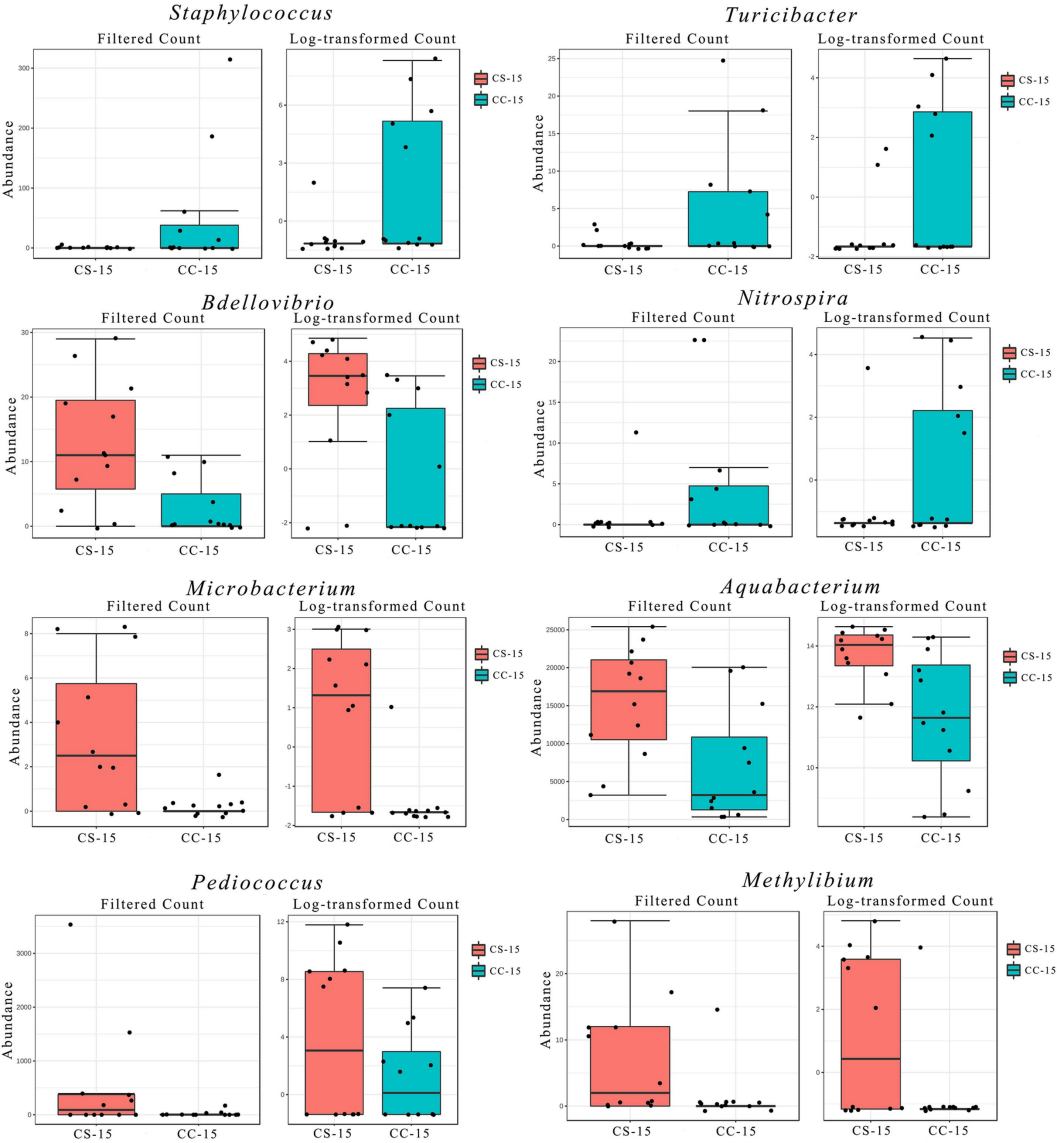
Filtration and analysis of the differential microbes in the yolk. Filtered count and log-transformed count of the differential microbes between the two groups. CC represents commercial broiler breeder eggs without eggshell surface sterilization, CS represents commercial broiler breeder eggs with eggshell surface sterilization, and the number represents incubation days.

**Figure 4:**
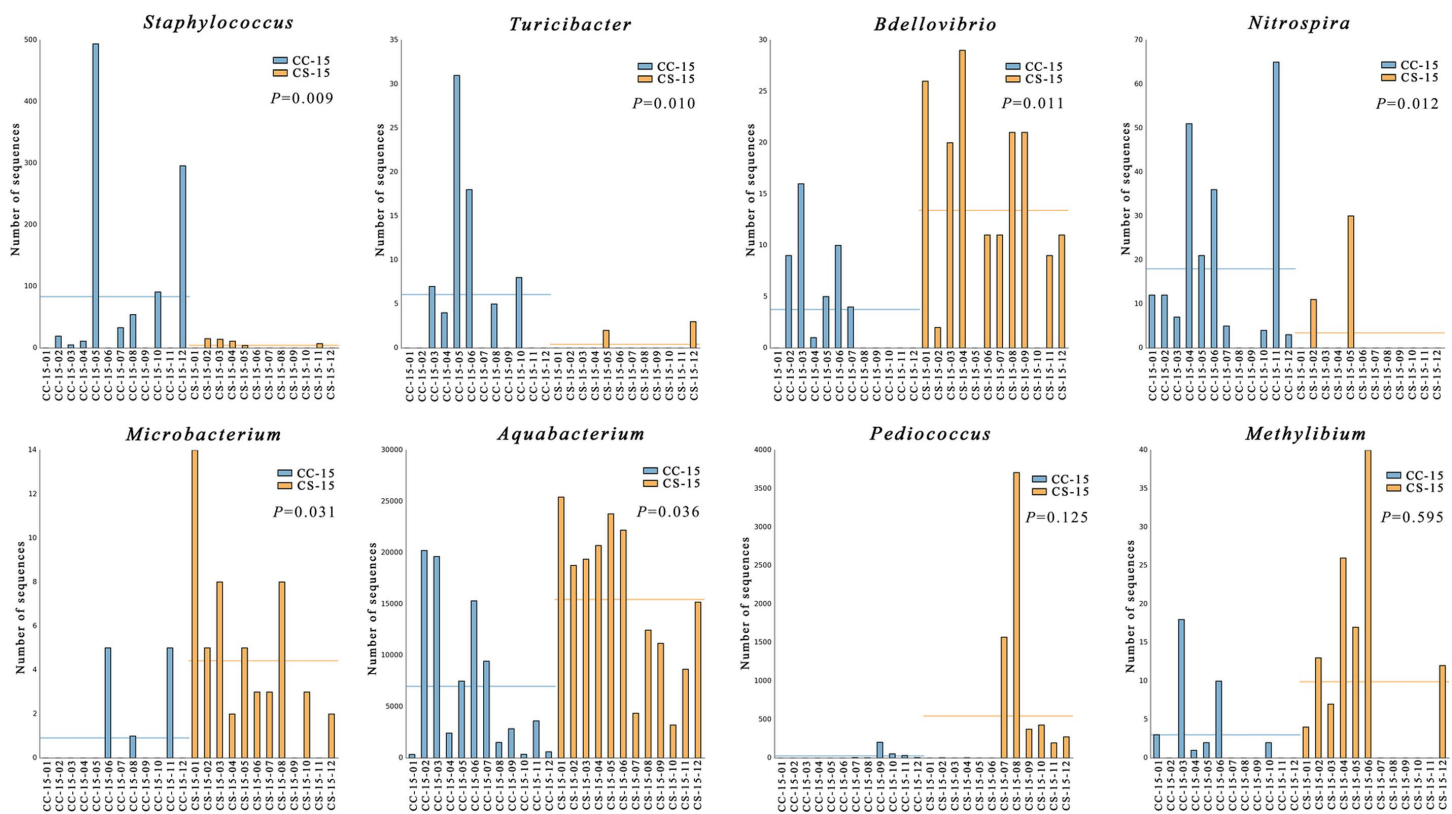
Analysis and comparison of the differential microbes in each replicate. CC represents commercial broiler breeder eggs without eggshell surface sterilization, CS represents commercial broiler breeder eggs with eggshell surface sterilization, and the number represents the number of samples.

**Figure 5:**
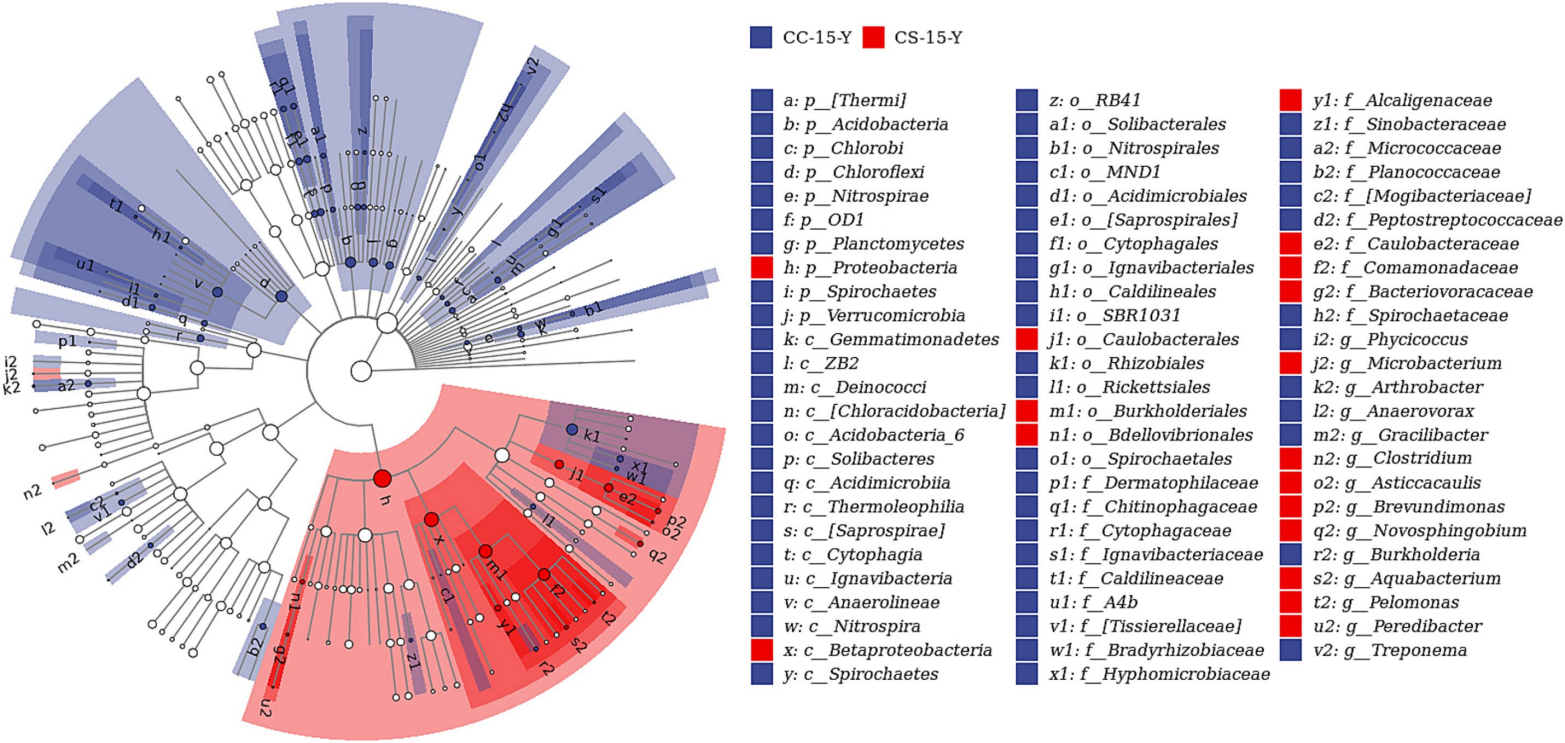
LEfSe analysis of the yolk microbiota in fertile eggs between the sterilized group and unsterilized group (E15). CC represents commercial broiler breeder eggs without eggshell surface sterilization, CS represents commercial broiler breeder eggs with eggshell surface sterilization, Y represents yolk, and the number represents incubation days.

In the previous analysis, we found that the effect of eggshell sterilization before incubation on the yolk microbiota was most pronounced at E15, so we further filtered and analyzed the differential yolk microbes at this time point. We finally filtered out eight major differential microbiota between group CS and group CC, namely, *Staphylococcus*, *Turicibacter*, *Bdellovibrio*, *Nitrospira*, *Microbacterium*, *Aquabacterium*, *Pediococcus*, and *Methylibium* (Figures 3, 4). The number of sequences of *Staphylococcus* (*p* = 0.009, *n* = 24), *Turicibacter* (*p* = 0.010, *n* = 24), and *Nitrospira* (*p* = 0.012, *n* = 24) in the yolk of the fertile eggs without surface sterilization was higher than that of sterilized eggs, while the sequence number of *Bdellovibrio* (*p* = 0.011, *n* = 24), *Microbacterium* (*p* = 0.031, *n* = 24), *Aquabacterium* (*p* = 0.036, *n* = 24), *Pediococcus* (*p* = 0.125, *n* = 24), and *Methylibium* (*p* = 0.595, *n* = 24) was lower in the yolk of the fertile eggs without surface sterilization than that in the yolk of sterilized eggs. In addition, LEfSe analysis of the yolk microbiota at different developmental stages showed that the variety of landmark microbiota in the yolk of the unsterilized eggs was higher than that of sterilized eggs at E7, E15, and E21 (Figure S4), especially at E15 (Figure 5). At E15, landmark microbiota at the genus level in the yolk of group CS mainly included *Microbacterium*, *Clostridium*, and *Asticcacaulis*, while that of group CC mainly included *Phycicoccus*, *Arthrobacter*, *Anaerovorax*, *Gracilibacter*, and *Burkholderia* (Figure 5).

### Identification of *Staphylococcus*

We performed 16S rDNA full-length sequencing to identify the species of *Staphylococcus* isolated from the yolk by selective media (Figure S3). The results showed that there were eight species of *Staphylococcus saprophyticus* and one species of *Staphylococcus pseudoxylosus*, and the sequence matches were greater than 99.86% (Table 1).

## Discussion

To date, studies on the microbiota in broilers have mainly focused on the post-hatch period, while studies on the presence and changes of microbiota in fertile eggs during embryogenesis are uncommon (31–33). Early studies in our laboratory found that the egg yolk is rich in the microbiota during embryogenesis, and the microbiota plays an important role in the utilization of yolk nutrients by chicken embryos. However, it is still unknown what impacts the composition and abundance of the yolk microbiota during embryogenesis. Previous studies have found a close relationship between the microbiota in fertile eggs and the microbiota in the oviduct and cloaca of the maternal hens (32). The allantoic membrane of the chicken embryo functions as a gas exchange with the outside during the embryonic development stage (34). This result indicated that the fertile egg is not an airtight environment; the microbes on the eggshell surface may enter the fertile egg through the eggshell and the allantoic membrane. So, we speculated whether the microbiota on the surface of the eggs would affect the microbiota in eggs.

Numerous studies have concluded that in livestock and poultry production, favorable physical conditions of newborn animals play a key role in the subsequent production performance and organ development (35, 36). For specific species of oviparous animals such as chickens, the main source of nutrients for embryonic development is the egg yolk. The ratio of the chicken’s weight at hatching to the initial egg weight of the fertile eggs reflects the developmental status of the chicken during embryogenesis (37). Here, we found that the ratio of chicken hatch weight to initial egg weight was significantly higher in the eggshell surface sterilization group than in the unsterilized group; however, a significant reduction in yolk microbial diversity was found in our results in the eggshell sterilized group compared to the control group. The microbiota in the gut of newborn animals has a variety of roles, such as the activation of the immune system (38) and the establishment of microecological homeostasis (39). In general, a richer microbial composition had a positive effect on the promotion of these functions (40).

Notably, the results showed that the differential microbes enriched in the yolk of unsterilized fertile eggs were mainly pathogenic microbes, among which *Staphylococcus* was the most abundant. Subsequently, we identified that the *Staphylococcus* isolated from the egg yolk was mainly *Staphylococcus saprophyticus* (Table 1). Infection with pathogenic bacteria can dramatically reduce the health status of animals, and the mobilization of the immune system caused by infection leads to a substantial loss of nutrients, which would negatively affect the production performance of animals (41). A comparison of the NCBI databases revealed that *Staphylococcus* is associated with the appearance of many diseases, and the same is true for *Turicibacter*. A study on mice (*Genus mus*) also found that *Turicibacter* is closely related to metabolic diseases (42). Therefore, we speculated that the better growth and development of chicken embryos after eggshell surface sterilization of fertile eggs might be due to the reduction of infection by *Staphylococcus saprophyticus*, *Turicibacter*, and other pathogenic microbes. It is worth mentioning that no beneficial bacteria were found among the differential bacteria, which may be related to the very low relative abundance of beneficial bacteria in the cloaca and the environment, and the sterilization treatment of the eggshell surface would kill the beneficial bacteria as well. If the presence of pathogenic microbes in the eggshell is avoided at the source by improving disease eradication in broiler breeders and hatchery management, rather than relying on additional disinfection treatments on the eggshell surface, could pathogenic microbial infections be avoided while maintaining the rich microbiota within the fertile eggs? This could be an important research direction to improve the health of newly hatched birds. At the time of chicken hatching (E21), the diversity indices in both the yolk microbiota and the intestinal microbiota of the embryos showed an increasing trend, regardless of surface sterilization. This may be mainly due to the direct contact between the chicken and the external environment at this time.

Our results showed that sterilization of the eggshell surface does affect the yolk microbiota and intestinal microbiota of chicken embryos during incubation. At E15, the yolk of fertile eggs in group CS with surface sterilization had a significantly higher abundance of Proteobacteria and a lower abundance of Firmicutes than that in group CC without surface sterilization. There was no such significant difference at other incubation time points. The allantoic membrane of a chicken embryo is immature enough to exchange gas with the outside in the early embryonic stage, while from the middle stage of incubation, the allantoic membrane gradually matures and the chicken embryo develops rapidly. During this stage, the chicken embryo efficiently used the yolk nutrients and conducted adequate gas exchange with the outside through the allantoic membrane (37), which may be the main reason for the abundance changes of Proteobacteria and Firmicutes in the yolk. Moreover, we found that the changes in the yolk microbiota during incubation were mainly concentrated in Firmicutes and Bacteroidetes at the phylum level. Previous publications have indicated that the ratio of Firmicutes to Bacteroidetes may be associated with nutrient absorption and weight gain (43–45). Interestingly, the relative abundance of all yolk microbiota in the fertile eggs without eggshell surface sterilization was higher than that of the sterilized ones at the phylum level, except for Proteobacteria. Such findings corroborated the previous results on microbial diversity and also suggested that sterilization of the eggshell surface had a noticeable effect on the yolk microbiota. *Aquabacterium* is a common microbe in the liquid phase environment, and the relative abundance of *Aquabacterium* in the yolk was always at the highest level during incubation. A recent study found that *Aquabacterium* may have a close relationship with the energy metabolic processes of the organism (46). Yolk is the main source of energy material during embryogenesis (47), so the presence of *Aquabacterium* may be associated with the development of chicken embryos. Moreover, *Aquabacterium* has generally been considered a potentially pathogenic microbe in previous studies (48, 49). Thus, we speculate that *Aquabacterium* may be related to the formation of the immune system during embryogenesis, and the exact relationship needs to be further studied. At the genus level, the gut microbiota of chicken embryos was most abundant in *Prevotella*, *Bacteroides*, and *Faecalibacterium* were most abundant in the gut microbiota of chicken embryos in both groups at E21. Earlier studies have shown that the fluctuation of *Prevotella* and *Faecalibacterium* in the microbiota of patients is related to metabolic disorders and inflammation, such as non-alcoholic steatohepatitis and adiposity (50–52). *Bacteroides* have been reported to decompose carbohydrate nutrients (53). Recent studies have found that these microorganisms are also closely related to the development of chickens (54, 55). The metabolic rates of chicken embryo liver and yolk sac are very active due to the vast consumption of lipids to supply energy and embryo growth during embryogenesis (56–58). At the late stage of embryonic development (approximately embryonic days 17–21), the yolk sac is withered gradually, and the function of nutrient absorption is degraded. Meanwhile, the remaining yolk directly enters the embryonic intestine through the yolk stem, and the nutrient absorption capacity of the intestine is increased gradually (59). The above may also be an important reason for the changes in the abundance of *Prevotella, Bacteroides*, and *Faecalibacterium* in the intestine. LEfSe analysis allowed us to identify the landmark microbiota between the fertile eggs with and without eggshell sterilization. The results showed that the number of landmark microbiota in the unsterilized group was higher than that in the sterilized group (Figure 5 and Supplementary Figure S4), which also verified the results of the microbial diversity analysis in the previous section. Moreover, we found that the landmark microbes identified by LEfSe analysis were also the differential microbes screened in the previous section, indicating that the presence of pathogenic microbes in the yolk was indeed related to whether the eggshell surface was sterilized or not.

In conclusion, sterilization of the eggshell surface before incubation can effectively reduce the diversity of microbiota in the yolk and reduce infection with pathogens such as *Staphylococcus saprophyticus*. The reduction of microbial diversity in eggs may be unfavorable to the activation of the embryonic immune system and the establishment of early intestinal microecological homeostasis. Therefore, the preferred way to reduce the infection with pathogenic microbes in fertile eggs is through the eradication of pathogens in the breeder hens and the improvement of hatchery management.

## Data Availability

The datasets presented in this study can be found in online repositories. The names of the repository/repositories and accession number(s) can be found at: https://www.ncbi.nlm.nih.gov/, SRR15221364.

## Footnotes

^1^ https://www.genescloud.cn
