# Supplementary material for: Effects of surface sterilization of fertile eggs on the yolk microbiota during the chicken embryo development
Source: Front Vet Sci. 2024 Nov 27;11:1493415. doi: 10.3389/fvets.2024.1493415 (PMC11631918; doi:10.3389/fvets.2024.1493415)
Supplement: Supplementary file 1 [file Data_Sheet_1.docx]

**Table S1. The relative abundance of microbiota in the yolk of fertile eggs at E15 (At the phylum level, %).**

| Item | Groups | | *P*-value |
| --- | --- | --- | --- |
|  | CC-15-Y | CS-15-Y |  |
| Proteobacteria | 49.65 ± 7.00^b^ | 78.53 ± 3.65^a^ | 0.003 |
| Firmicutes | 28.29 ± 7.36 | 13.67 ± 3.95 | 0.091 |
| Bacteroidetes | 9.76 ± 3.80 | 4.27 ± 0.93 | 0.184 |
| Actinobacteria | 5.50 ± 1.55 | 2.34 ± 0.68 | 0.081 |
| Cyanobacteria | 1.94 ± 1.10 | 0.20 ± 0.04 | 0.143 |
| Chloroflexi | 1.31 ± 0.57 | 0.14 ± 0.05 | 0.066 |
| Acidobacteria | 0.65 ± 0.26 | 0.14 ± 0.06 | 0.077 |
| Chlorobi | 0.34 ± 0.17 | 0.00 ± 0.00 | 0.072 |
| Thermi | 0.29 ± 0.12 | 0.04 ± 0.01 | 0.062 |
| Gemmatimonadetes | 0.22 ± 0.09 | 0.07 ± 0.02 | 0.114 |
| Spirochaetes | 0.25 ± 0.19 | 0.00 ± 0.00 | 0.203 |
| WS6 | 0.24 ± 0.19 | 0.00 ± 0.00 | 0.214 |
| Verrucomicrobia | 0.15 ± 0.05^a^ | 0.03 ± 0.01^b^ | 0.027 |
| TM7 | 0.15 ± 0.06 | 0.02 ± 0.01 | 0.054 |
| OD1 | 0.16 ± 0.09 | 0.00 ± 0.00 | 0.092 |

In the same row, values with no letter or the same letter superscripts means no significant difference (*P* > 0.05), while with different letter superscripts mean significant difference (*P* < 0.05).

**Table S2. The relative abundance of microbiota in the yolk of fertile eggs at E15 (At the genus level, %).**

| Item | Groups | | *P*-value |
| --- | --- | --- | --- |
|  | CC-15-Y | CS-15-Y |  |
| *Aquabacterium* | 19.48 ± 6.05^b^ | 43.11 ± 6.01^a^ | 0.012 |
| *Clostridium* | 6.45 ± 4.33 | 5.57 ± 2.56 | 0.845 |
| *Lactococcus* | 8.96 ± 3.41 | 1.87 ± 1.73 | 0.082 |
| *Acidovorax* | 3.48 ± 1.21 | 6.12 ± 0.93 | 0.100 |
| *Lactobacillus* | 6.08 ± 4.18 | 2.75 ± 1.18 | 0.443 |
| *Novosphingobium* | 2.37 ± 0.92 | 3.72 ± 0.84 | 0.288 |
| *Pseudomonas* | 1.40 ± 0.42 | 3.67 ± 1.35 | 0.130 |
| *Flavobacterium* | 2.27 ± 0.55 | 2.65 ± 0.72 | 0.681 |
| *Azospirillum* | 1.70 ± 0.51 | 2.73 ± 0.46 | 0.146 |
| *Enterobacter* | 0.67 ± 0.35 | 3.29 ± 1.30 | 0.073 |
| *Bifidobacterium* | 2.52 ± 1.55 | 0.26 ± 0.06 | 0.173 |
| *Bacillus* | 2.19 ± 1.90 | 0.43 ± 0.18 | 0.366 |
| *Burkholderia* | 2.54±1.65 | 0.07±0.02 | 0.163 |
| *Leuconostoc* | 1.29±0.67 | 0.34±0.15 | 0.193 |
| *Chryseobacterium* | 0.47±0.21 | 1.15±0.60 | 0.301 |

In the same row, values with no letter or the same letter superscripts means no significant difference (*P* > 0.05), while with different letter superscripts mean significant difference (*P* < 0.05).


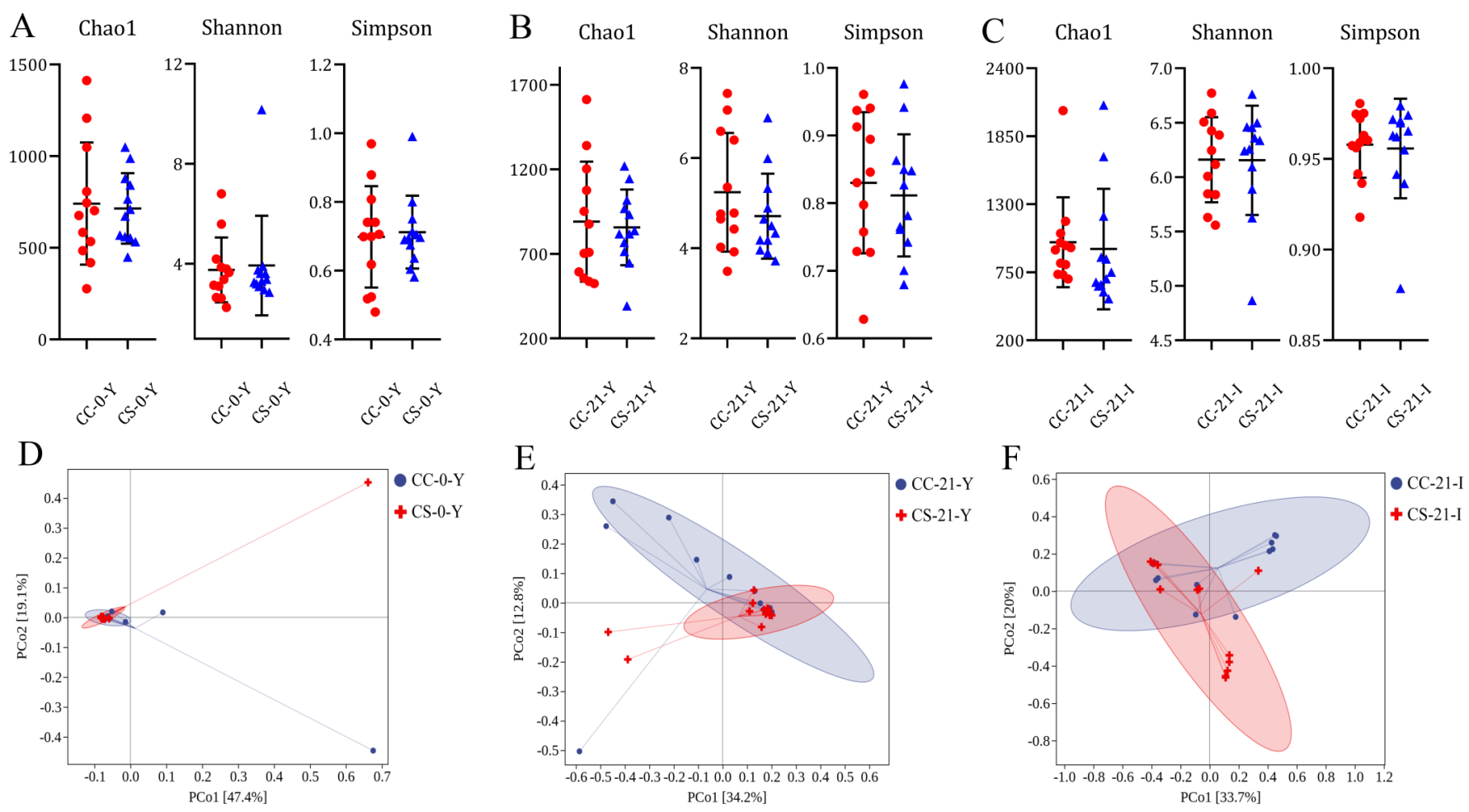


**Figure S1. Effect of eggshell surface sterilization of fertile eggs before incubation on microbial diversity in the yolk and intestine.** CC in the picture represents commercial broiler breeder eggs without eggshell sterilization, CS in the picture represents commercial broiler breeder eggs with eggshell sterilization, Y represents yolk, I represents intestine, and the number represents the days of incubation. (A, B, C) The alpha diversity of yolk microbiota in the yolk at E0 (A), E21 (B) and in the intestine at E21 (C). (D, E, F) The beta diversity of yolk microbiota in the yolk at E0 (D), E21 (E) and in the intestine at E21 (F).


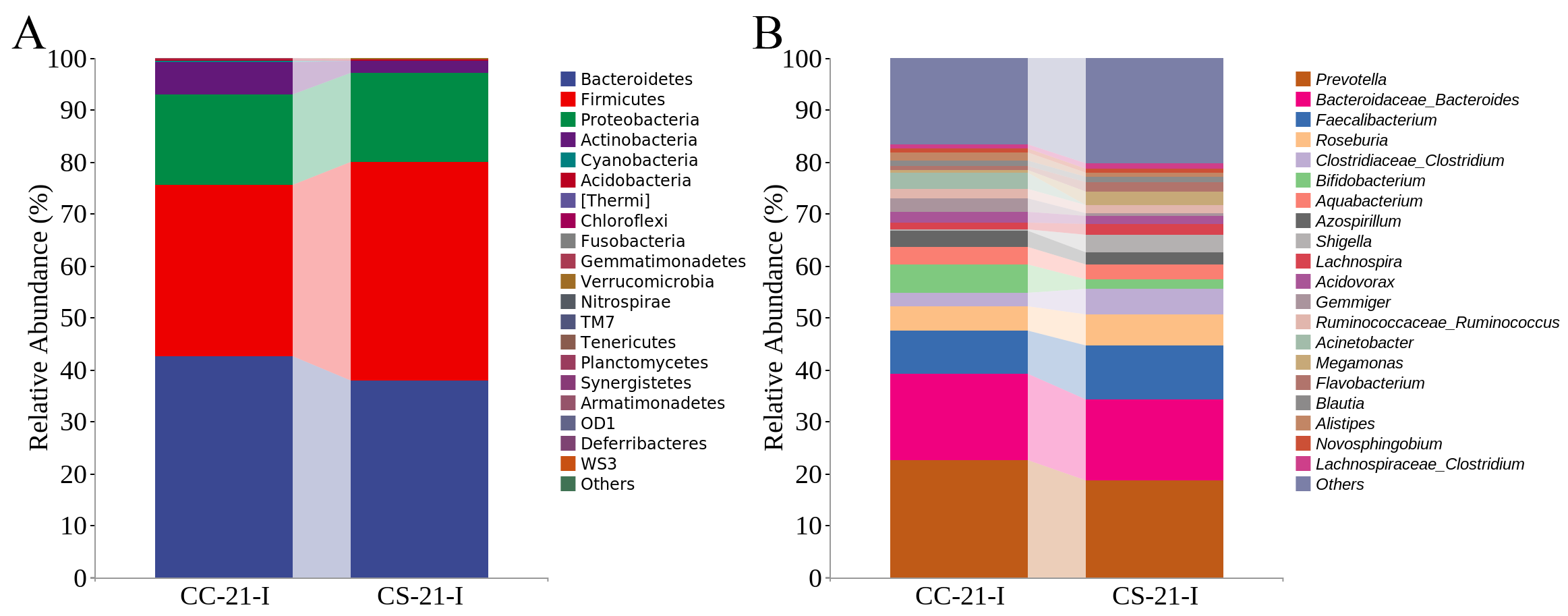


**Figure S2. The relative abundance of intestinal microbiota in eggshell surface sterilized and unsterilized fertile eggs at E21.** CC in the picture represents commercial broiler breeder eggs without eggshell sterilization, CS in the picture represents commercial broiler breeder eggs with eggshell sterilization, I represents intestine, and the number represents the days of incubation. (A) At the phylum level. (B) At the genus level.


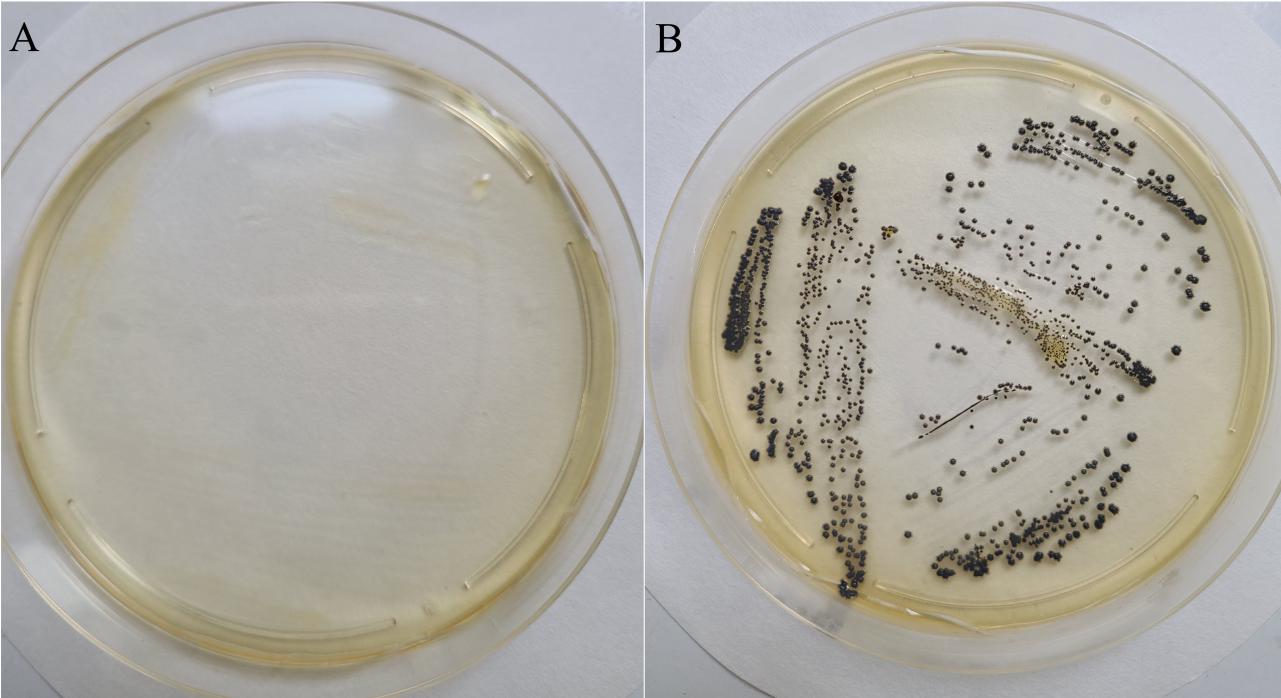


**Figure S3. The plate culture of *Staphylococcus* (E15).** (A) Eggshell surface sterilization group. (B) Eggshell surface unsterilization group.


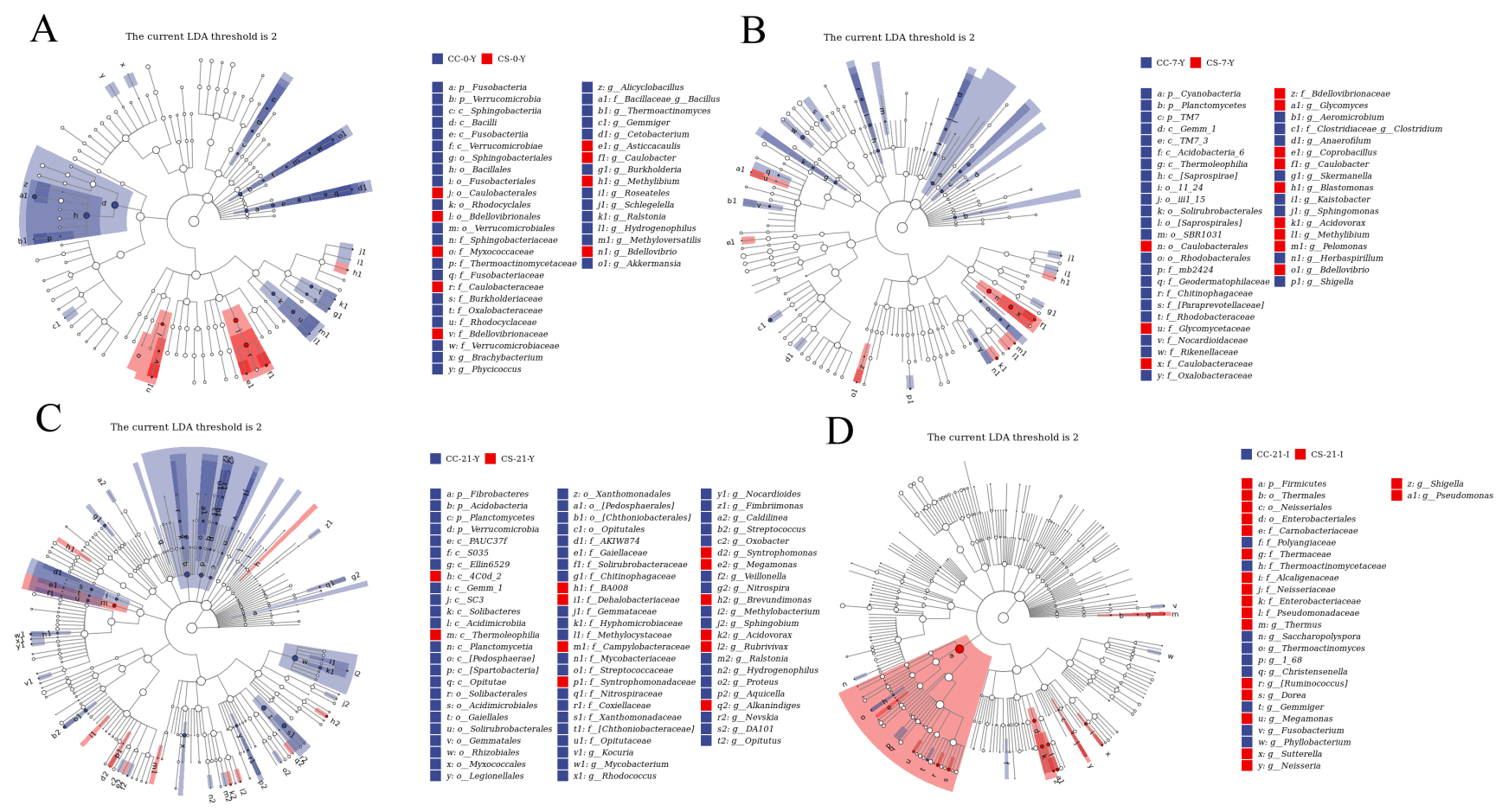


**Figure S4. LEfSe analysis of the yolk microbiota and intestinal microbiota in fertile eggs between the sterilized group and unsterilized group at different embryonic stages.** CC in the picture represents commercial broiler breeder eggs without eggshell sterilization, CS in the picture represents commercial broiler breeder eggs with eggshell sterilization, Y represents yolk, I represents intestine, and the number represents the days of incubation.
